# Isolation of Biologically Active Compounds from *Cannabis sativa* L. Inflorescences by Using Different Extraction Solvents and Evaluation of Antimicrobial Activity

**DOI:** 10.3390/antiox12050998

**Published:** 2023-04-25

**Authors:** Dovilė Motiejauskaitė, Sana Ullah, Algimanta Kundrotaitė, Renata Žvirdauskienė, Aušra Bakšinskaitė, Karolina Barčauskaitė

**Affiliations:** 1 Lithuanian Research Centre for Agriculture and Forestry, Institute of Agriculture Instituto al. 1, Akademija, LT-58344 Kėdainiai District, Lithuania; 2 Department of Food Science and Technology, Kaunas University of Technology, LT-50254 Kaunas, Lithuania

**Keywords:** *Cannabis sativa* L., polyphenolic compounds, radical scavenging activity, CBD, antimicrobial activity

## Abstract

Hemp inflorescences are a source of vital compounds, including phytocannabinoids and other biologically active compounds. Various methods are adapted for the extraction of these vital compounds such as the use of different organic solvents. This study aimed to assess the comparative extraction potential of three different solvents: deionized water, 70% methanol (MeOH), and 2% Triton X-100, for phytochemicals in hemp inflorescences. Spectrophotometric techniques were applied to investigate the total amount of polyphenolic compounds (TPC), total flavonoids contents (TF), phenolic acids (TPA), and radical scavenging activity (RSA) in hemp extracts obtained using different polarity solvents. Gas chromatography-mass spectrometry was used for cannabinoids and organic acids quantitative analysis. In the results, MeOH showed a better affinity for the recovery of TFC, TPA, and RSA in comparison to Triton X-100 and water. However, Triton X-100 performed better for TPC with 4-folds and 33% turnover compared to water and MeOH, respectively. Six cannabinoids (CBDVA, CBL, CBD, CBC, CBN, and CBG) were identified in hemp inflorescence extracts. The maximum determined concentration was as follows: CBD > CBC > CBG > CBDVA > CBL > CBN. Overall, fourteen organic acids were identified. Hemp inflorescence extracts obtained using 2% Triton X-100 showed an effect on all tested strains of microorganisms. Methanolic and aqueous extracts had antimicrobial activity against seven tested strains. On the other hand, the inhibition zones were wider for methanolic extracts compared to aqueous ones. Hemp aqua extract with antimicrobial activity might be used in various markets where toxic solvents are unwanted.

## 1. Introduction

Hemp (*Cannabis sativa* L.) is an herbaceous plant belonging to the family *Cannabaceae* that has been used by humans for 5000 years [1]. The genus *Cannabis* is cultivated for different purposes, including fiber, food, and medicine [2]. It is promising that the whole above-ground part, from stem to flower, along with the seeds of industrial hemp could be processed for further use [3,4]. Especially the morphological parts, such as the inflorescence, have significant importance due to the presence of potentially biologically active compounds [5,6].

Furthermore, industrial hemp inflorescence possessed a wide composition of biologically active compounds called secondary metabolites, including phytocannabinoids, terpenes, phenolic compounds, and fatty acids [7,8,9]. Over 100 cannabinoids have been identified; most of them are found in female inflorescences [10]. However, only a few of them are found in larger quantities such as cannabidiol (CBD), cannabigerol (CBG), cannabinol (CBN), Δ9-tetrahydrocannabinol (Δ9-THC), and cannabichromene (CBC). The concentration of cannabinoids mainly depends on the genetic properties of the plant; however, variety, age, environmental conditions (nutrition, humidity, light level), harvest time, and storage conditions also play an important role [11].

Presently, CBD is gaining a lot of attention due to its pharmacological properties, such as pain relief, anti-anxiety, relaxation promotion, anti-nausea, anti-psychotic, anti-inflammation, immunomodulatory, and antimicrobial activity [11,12]. The concentration of CBD in hemp varies from 1790 µg g^−1^ in leaves to 8590 µg g^−1^ in flowers on a dry weight basis [11]. Furthermore, it was determined that wild hemp essential oil contains a significant amount of CBD, with a concentration ranging from 6.9% to 52.4% of the total oil [13]. Information on other cannabinoids is lacking because often only CBD, THC, or total cannabinoid levels are studied. Other types of cannabinoids are found in low quantities and are called minor cannabinoids [14].

Another very important group of biologically active compounds is terpenes. More than 120 terpenes have been identified in hemp [12]. Terpenes are well known for their anti-cancer, anti-heartburn and gastroesophageal reflux, antimicrobial, antiviral, antihyperglycemic, antihyperglycemic, anti-inflammatory, and immunomodulatory properties [15]. The terpene profile of cannabis depends on a few key factors: extraction method, growing conditions, and plant age. Terpenes content ranges from 0.125% to 0.278% in leaves and from 1.283% to 2.141% in the inflorescence on a dry weight basis [12]. Monoterpenes are usually the dominant terpenes, with concentrations of 3.1 mg g^−1^ to 28.3 mg g^−1^ of dry weight [11,12]. Sesquiterpenes take second place depending on the detected amount, which varies from 0.5 mg g^−1^ to 10.1 mg g^−1^ of inflorescence dry weight [11].

Polyphenols also play a very important role when talking about biologically active compounds in hemp tissue. Flavonoids, phenolic acids, and lignans are the most significant compounds possessing influential antimicrobial and biological activities [16]. Phenols are powerful antioxidants that help to neutralize the damaging effects of free radicals and reduce oxidative damage in plants. They also have anti-inflammatory, anti-diabetic, cardioprotective, neuroprotective, antitumor, and antiaging effects [17]. Total polyphenolic content in inflorescences ranges from 10.51 to 52.58 mg GAE g^−1^ of dry weight. Flavonoids account for about 80% of the total amount of phenolic compounds in hemp inflorescences, with a concentration range of 222.7 to 454.0 mg kg^−1^ dry weight. Furthermore, phenolic acids make up 16.6% to 19.1% of the total amount of phenolic compounds and range from 65.4 to 123.62 mg kg^−1^ dry weight [16].

Although various methods are adopted for the extraction of biologically active compounds. Among these methods, classical approaches are commonly practiced and use a variety of organic solvents, including methanol, ethanol, acetone, ether, ethyl acetate, and hydrocarbons [2,12,18]. Except for classical approaches, other emerging techniques are also used, which include green methods such as micelle-mediated extraction methods with surface-active compounds (sodium dodecyl sulphate (SDS), cetyltrimethylammonium bromide (CTAB) or Tritones (X-100 or X-114), Tweens (20 or 80), and Genapol X-080), ultrasound extraction, supercritical fluid extraction, microwave-assisted extraction, etc. [19,20]. Comparing these green methods with classical ones, their advantages are that they are often environmentally friendly, achieve a higher extraction yield with a shorter extraction time, and possess higher biological activity [19,21].

Based on the literature, ethanol is the most commonly used solvent in the extraction of hemp’s biologically active compounds. According to the Food and Drug Administration (FDA), ethanol is less toxic compared to other solvents [12]. In addition to ethanol, methanol extraction yields a higher concentration [22]. It was found that extraction with methanol yielded the highest amount of phenolic compounds (0.890 mg GAE g^−1^) in the pure hemp leaves compared to extraction with acetone, which resulted in a low amount (0.416 mg GAE g^−1^). The same results were observed for sugar beet leaves, in which methanol extraction showed the best results compared to chloroform, ethyl acetate, and petroleum ether solvents [18].

Surfactants are a promising alternative to organic solvents, as they are safe, flameproof, non-toxic, and easy to use. Organic solvents show better yields of phenolic compounds, but with minor differences. After examining *Taraxacum officinale* extracts, it was found that the total amount of phenolic compounds in acetone extracts is 23.4% higher compared to Triton X-100 extract [20]. However, another study stated that surfactants were found to have the highest extraction efficiency compared to methanolic solvents. This is explained by the fact that surface-active compounds have polar and non-polar properties due to their structure [23].

Thereby, this experiment aimed to (i) compare the potential of different polarity solvents on the recovery of biologically active compounds from industrial hemp inflorescences and (ii) investigate the extract’s antimicrobial activity against different bacterial strains (Gram-negative and Gram-positive) and yeast-like fungi.

## 2. Materials and Methods

### 2.1. Chemicals

Methanol was obtained from Sigma-Aldrich, St. Quentin, France. Triton X-100 was purchased from PanReac AppliChem ITW Reagent, Barcelona, Spain. Aluminum chloride anhydrous (pure for analysis) and sodium nitrite (pure for analysis) were purchased from Chempur, Piekary Śląskie, Poland. Caffeic acid ≥ 98% and rutin ≥ 97% were obtained from Acros Organics, Berlin, Germany. Folin–Ciocalteu reagent, sodium carbonate, and hydrochloric acid were obtained from Sigma-Aldrich, Schnelldorf, Germany. Ethyl acetate and pyridine were purchased from VWR International S.A.S, Briare, France. Magnesium sulphate was obtained from VWR International, Leuven, Belgium. Resveratrol (5-[(E)-2-(4-hydroxyphenyl)vinyl]benzene-1,3-diol) was purchased from Apollo Scientific, Bredbury, UK. BSA 95–100% (N,O-bis-trimethylsilyl-acetamide) was obtained from Macherey-Nagel, Dueren, Germany. Sodium hydroxide was purchased from Carl Roth, Karlsruhe, Germany. DPPH (1,1-Diphenyl-2-picrylhydrazyl free radical) was obtained from TCI EUROPE, Zwijndrecht, Belgium. For all dilutions and extractions, bidistilled water was obtained using the distillation apparatus from Thermo Scientific (Waltham, MA, USA).

### 2.2. Preparation of Plant Extracts

The objects of this research were inflorescences of *Cannabis sativa* L., variety *Felina 32*, collected from the Lithuanian Research Centre for Agriculture and Forestry experimental fields during the 2020 and 2021 growing seasons. Experimental treatments were placed in a randomized block with four replicates. The total area of each experimental plot was 27 m^2^ (9 × 3 m). The plants were fertilized with N_150_P_60_ K_60._ The phosphorus (in the form of superphosphate) and potassium (in the form of potassium chloride) were distributed manually in particular fields of treatment three weeks after the sowing. Fertilization by nitrogen was split, and the first fertilization of 70 kg/ha of nitrogen (in the form of ammonium nitrate) was applied after the plants’ germination vegetation and supplemented by 80 kg/ha two weeks later. The plants from 0.25 m^2^ (0.5 × 0.5) were harvested at the full maturity stage and divided into morphological parts (stems, leaves, and inflorescences). The dried inflorescences of fibrous hemp were crushed with a grinder (Retch ZM 200, Haan, Germany) and used for the analysis.

#### 2.2.1. Preparation of Plant Extracts for Total Polyphenolic Compounds, Total Flavonoids, Total Phenolic Acids, Radical Scavenging Activity Analysis and Antimicrobial Testing

Approximately 1 g of raw industrial hemp inflorescence material was mixed with deionized water, 70% methanol solution, or 2% Triton X-100 solution, respectively, at a ratio of 1:10. The test tube with plant material and the solvent was thoroughly mixed using a vortex (IKA MS3, Wilmington, NC, USA) and treated in an ultrasonic bath for 60 min at ambient temperature. Then, the extracts were filtered using a paper filter at 90 g/m^2^ and stored at +4 °C until analysis.

#### 2.2.2. Preparation of Plant Extracts for Phytocannabinoids Analysis

Approximately 0.25 g of raw material was mixed with methyl alcohol at a ratio of 1:20 and exposed to an ultrasonic bath for 60 min at 28 °C. The extracts were filtered using paper filters into tubes, and before the analysis, the extracts were filtered through a 0.45 µm nylon filter.

#### 2.2.3. Preparation of Plant Extracts for Organic Acids Determination

Organic acids analysis was performed using a standard procedure as described earlier [24]. For this, 500 mg of air-dried industrial hemp inflorescence powder was mixed with 10 mL of 2 M NaOH and 0.1 mg resveratrol as an internal standard and vortexed for one min (IKA MS3, USA). Then, the mixture was macerated in an ultrasonic bath for 15 min at room temperature. Thereafter, the extract was filtered and washed twice with 5 mL ethyl acetate, then acidified with 1 M HCl to pH 2, and again washed twice with 2.5 mL ethyl acetate. The acetate fraction was transferred to a separate tube and washed twice with 2.5 mL of deionized water. The organic phase was dried with MgSO_4_, filtered through a 0.45 µm nylon filter, and evaporated to dryness using nitrogen flow. The sample was derivatized with 750 µL of pyridine and 150 µL of BSA silylation reagent by maintaining it in a heating block at 60 °C for 20 min.

### 2.3. Determination of Total Phenolic Compounds Content (TPC)

The TPC was determined by the spectrophotometric method at a wavelength of 760 nm, following the standard procedure as described earlier [25]. Test samples were obtained by mixing 0.1 mL of the extract with 2.5 mL of bidistilled water, 0.1 mL of Folin–Ciocalteu reagent, and 0.5 mL of a 20% Na_2_CO_3_ solution. A blank sample was prepared following the same procedure instead of extracting with different extraction solvents. The resulting solutions were incubated in the dark for 30 min. After that, their absorbance was measured using a spectrophotometer (Shimadzu, Kyoto, Japan). Thereafter, the concentration of TPC was calculated using the linear equation of the rutin calibration curve: y = 1.1038x − 0.1345.

### 2.4. Determination of Total Flavonoids Content (TFC)

The total amount of flavonoids was determined by the spectrophotometric method at a wavelength of 510 nm, following the standard procedure [25]. Test samples were obtained by mixing 1 mL of the extract with 0.3 mL of 5% NaNO_2_, adding 0.5 mL of 2% AlCl_3_ after 5 min, and adding 0.5 mL of 1 M NaOH after another 6 minThe sample for comparison was obtained in the same way, instead of using plant extracts different extraction solvents were used. The resulting solutions were incubated in the dark for 10 min. After that, their absorbance was measured using a spectrophotometer (Shimadzu, Japan). After that, the TFC was calculated using the linear equation of the rutin calibration curve: y = 2.39x + 0.4745.

### 2.5. Determination of Total Amount of Polyphenolic Acids (TPA)

The TPA was determined by the spectrophotometric method at a wavelength of 505 nm, following the standard procedure [25]. Test samples were obtained by mixing 0.5 mL of the extract with 1 mL of 0.5 M HCl, 1 mL of 8.5% NaOH, 1 mL of Arnow’s reagent, and 1.5 mL of bidistilled water. The sample for comparison was obtained in the same way, instead of using plant extracts different extraction solvents were used. The absorbance of samples was measured using a spectrophotometer (Shimadzu, Japan). The concentration of polyphenolic acids was calculated using the linear equation of the caffeic acid calibration curve: y = 0.1982x + 0.0393.

### 2.6. Antioxidant Activity

The antioxidant activity of the extracts was determined using the 2,2-diphenyl-2-picrylhy-drazyl (DPPH) free radical scavenging activity method. Briefly, 0.077 mL of extract was mixed with 3 mL of 6 × 10^−5^ M DPPH. The absorbance of the resulting faded pink or yellow solution was measured at 515 nm after 15 min of incubation in the dark. DPPH radical scavenging activity (%) was calculated using Formula (1):

(1) $$DPPH\;radical\;scavenging\;activity\;(\%)=\frac{Abs.of\;blank-Abs.of\;sample}{Abs.of\;blank}\times 100$$

### 2.7. Quantitative Analysis of Phytocannabinoids

Quantitative analysis of phytocannabinoids was performed using the Shimadzu GC-MS QP2010 system (Shimadzu, Japan). A 30 m long Rxi-5ms (Restek, Bellefonte, PA, USA) column was used for cannabinoids separation. Column thickness—0.25 µm; inner diameter—0.25 µm. Helium was used as the carrier gas. Gas chromatograph conditions: initial column temperature of 110 °C maintained for 2 min, then raised to 190 °C at a rate of 10 °C/min and maintained for 10 min; at a rate of 10 °C/min, the temperature was raised to 280 °C and held for 10 min. The total analysis time for one sample was 39 min. The temperature of the injector was 250 °C, the samples were inserted using an autoinjector (AOC-5000 Plus, Shimadzu, Japan), and the injection was performed by the split 1:10 method. An injection volume of 1 µL was used. The following mass spectrometer conditions were set: ion source temperature: 200 °C, interface temperature: 280 °C, solvent exit time: 2.5 min, and sample ionization energy: 70 eV. Scan speed: 1666; scan interval: start 35.00 *m*/*z*, end: 500.00 *m*/*z*. The obtained sample chromatograms were analyzed using GCMS solution (Shimadzu, Japan) software. The compounds were identified according to the mass-to-charge ratio by comparing the mass spectra of standard and identified compounds. The quantitative analysis was performed using the external standard method.

### 2.8. Quantitative Analysis of Organic Acids

The separation and identification of industrial hemp inflorescence organic acids composition were performed using gas chromatography-mass spectrometry (GC-MS-QP2010 Ultra) (Shimadzu, Japan). The percentage expression of the composition of the compound was calculated by considering all of the identified compounds. The GC device was equipped with a 30 m long Rxi-5ms (Restek, USA) column (30 m × 0.25 mm × 0.25 μm). The operating conditions of the GC were as follows: the initial temperature was 100 °C, then raised to 190 °C at a rate of 2.0 °C/min and, at a rate of 5 °C/min, raised to 300 °C and held for 25 min; the injector was held at 280 °C throughout the analysis; and helium was used as a carrier gas with a linear velocity of 44.2 cm s^−1^ at a flow rate of 1.41 mL per minute. Additionally, the MS operational conditions were as follows: ion source temperature 250 °C; interface temperature 250 °C; electron impact ionization at 70 eV; and scanning mode from 40 to 1000 *m*/*z* at a speed of 3333.

### 2.9. Antimicrobial Activity

Antimicrobial activity was determined using the agar well diffusion method against selected Gram-positive (*Staphylococcus aureus* ATCC 25923, *Bacillus cereus* ATCC 11778, *Bacillus subtilis* ATCC 6633, *Listeria monocytogenes* ATCC 13932, *Bacillus megaterium* ATCC 33085, *Enterococcus faecalis* ATCC 19433, *Micrococcus luteus* ATCC 9341) Gram-negative (*Salmonella enteritidis* ATCC 13076, *Escherichia coli* ATCC 8739, *Pseudomonas aeruginosa* ATCC 10145, *Salmonella typhimurium* ATCC 14028) bacteria and one yeast-like fungus (*Candida albicans* ATCC 10231). After 24 h of incubation, the bacterial suspension was prepared with sterile saline to 1.5 × 108 CFU/mL (turbidity = McFarland barium sulfate standard 0.5). Prepared PCA (plate count agar) medium was inoculated with fresh bacterial suspension under aseptic conditions so that the cell count in the medium would be approximately 1.5 × 107 CFU/mL and was poured into Petri dishes (a 90 mm diameter Petri dish requires approximately 12 mL of medium). After the medium solidified, wells with a diameter of 9 mm were made in each plate with a sterile tip, into which 40 μL of the tested hemp extracts were added. All experiments were performed in triplicate wells for each condition and repeated three times. Plates were incubated at 37 °C for 24 h. The antimicrobial activity of extracts was determined by measuring the inhibition zones at the two perpendicular diameters using a caliper.

The degree of bacterial and fungal susceptibility was evaluated based on the radius of the inhibition zone, which was calculated using Formula (2):

(2) $$Radius=\frac{Diameter\;of\;inhibition\;zone-Diameter\;of\;the\;well}{2}$$

As a negative control, sterile solvents (deionized water, 70% methanol solution (MeOH), and 2% Triton X-100 solution) were used. Three replicates at each concentration were performed.

### 2.10. Statistical Analysis

Results were presented as the mean of three replicates ± standard deviation. The statistics software package “STATISTICA 8.1” was used for the analysis of the collected data. A one-way analysis of variance (ANOVA) statistical package was used to evaluate the data scatter and determine statistically significant differences between the means. The essential differences between different extraction solvents’ recovery of biologically active compounds were evaluated using Fisher’s criterion at *p* ≤ 0.05.

## 3. Results

### 3.1. Spectrophotometric Results

**Figure 1:**
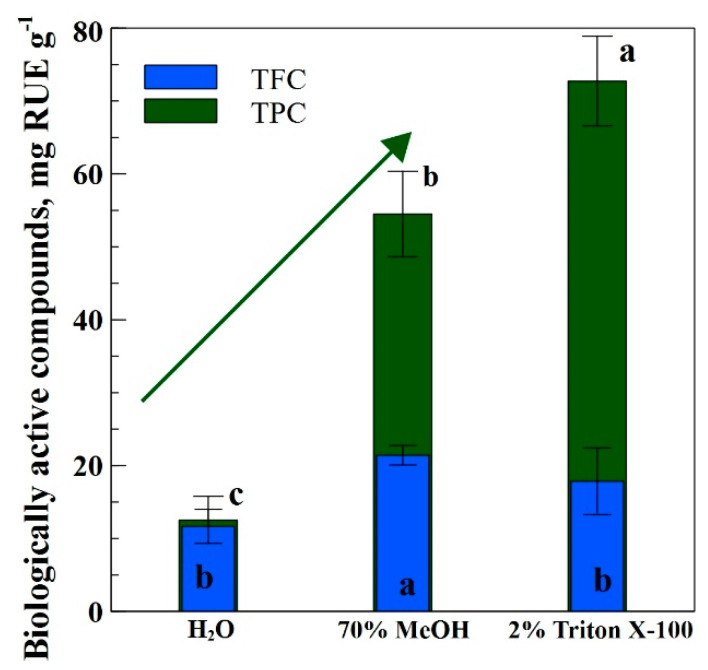
Amount of TFC and TPC extracted from industrial hemp inflorescence using different polarity solvents; different letters indicate the significant differences at *p* < 0.05. The green arrow in the figure demonstrates an increasing amount of TPC.

Figure 1 represents the effect of different solvents (H_2_O, 70% MeOH, and 2% Triton X-100) on biologically active compounds extracted from industrial hemp inflorescence. It was observed that extraction with Triton X-100 yields better total polyphenolic compounds (TPC) contents, with a value of 72.73 mg RUE g^−1^ dry weight. After Triton X-100, extraction with MeOH gave 54.49 mg RUE g^−1^ dry weight. However, extraction with H_2_O proved to be the least effective, with a value of 12.51 mg RUE g^−1^ dry weight only. Regarding the total flavonoid contents (TFC), extraction with MeOH yields better, with a maximum value of 21.41 mg RUE g^−1^ dry weight following extraction with Triton X-100 of 17.85 mg RUE g^−1^ dry weight. Extraction with H_2_O proved to be the least effective, with a value of 11.67 mg RUE g^−1^ dry weight only.

**Figure 2:**
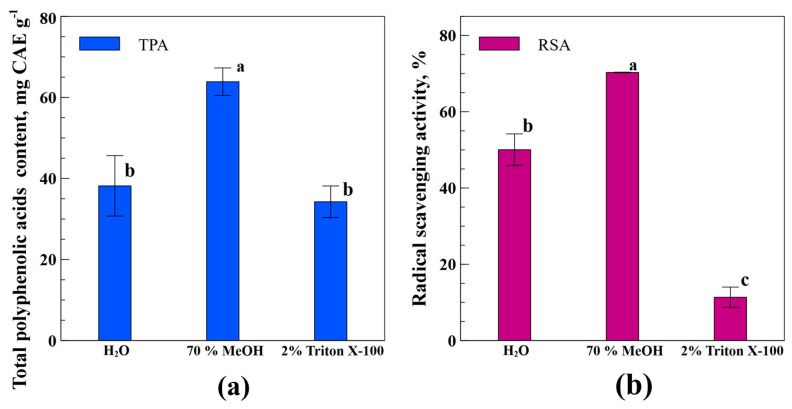
The amount of TPA (**a**) and RSA (**b**) in industrial hemp inflorescence extracts obtained using different polarity solvents; different letters indicate the significant differences at *p* < 0.05.

Figure 2a shows the effect of different solvents (H_2_O, 70% MeOH, and 2% Triton X-100) on total polyphenolic acids (TPA), and Figure 2b shows the radical scavenging activity (RSA) of industrial hemp inflorescence extracts. Extraction with MeOH yields better than other solvents regarding TPA, with a maximum value of 63.88 CAE g^−1^ dry weight (Figure 2a). Extraction with H_2_O showed 38.18 CAE g^−1^ dry weight, followed by Triton X-100 with the least yield of 34.26 CAE g^−1^ dry weight, but both treatments were statistically non-significant to each other. Likewise, for RSA, extraction with MeOH yields better than other solvents, with a maximum value of 70.3% (Figure 2b). Extraction with H_2_O showed RSA with a value of 50%; however, Triton X-100 extraction remained the least effective with an RSA yield of only 11.36%.

### 3.2. The Quantitative and Qualitative Analysis of Hemp Inflorescence

Table 1 shows the composition of cannabinoids determined in the methanolic extract of industrial hemp inflorescences. Five cannabinoids, including CBL, CBD, CBC, CBN, and CBG, and one in acid form, CBDVA, were determined. Among all, CBD occupied a significant portion (2.50% of a total value of 3.5% of cannabinoids). After CBD, CBC shows 0.333% of a total value of 3.5% of cannabinoids. Other constituents were followed by CBG (0.134%) > CBDVA (0.094%) > CBL (0.024%) > CBN (0.022%).

**Figure 3:**
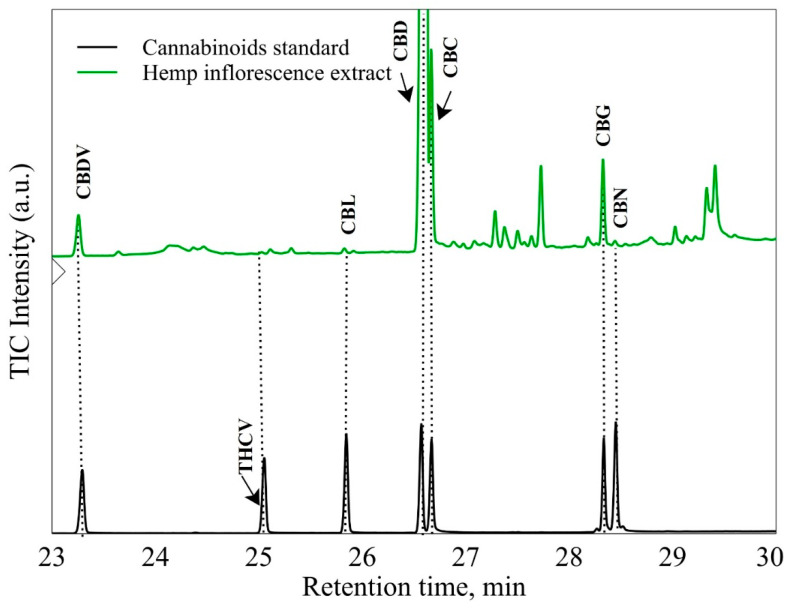
Chromatographic profile of cannabinoids standards and industrial hemp inflorescences methanolic extract.

The qualitative analysis of cannabinoids is presented in Figure 3. Industrial hemp inflorescence methanolic extracts were compared to standard solution retention times and individual compounds’ mass-to-charge ratio (*m*/*z*).

### 3.3. Organic Acids Composition in Hemp Inflorescence

The determined organic acids composition in hemp inflorescences is given in Table 2. Overall, fourteen different acids were identified in the tested hemp inflorescence samples. It was found that despite agrochemical conditions, the dominant acid is cannabidiolic acid, which ranged from 15.43% to 24.99% of the total amount of identified compounds. This result is justified by the fact that the hemp variety *Felina 32* is known as a cannabidiol-producing hemp type, and similar results were presented in previous works [14,26]. Two polyunsaturated fatty acids: linoleic acid and alfa-linolenic acid, were also found in significant amounts, averaging around 18.89 and 12.92, respectively. These acids are essential and extremely important for human health [27], as they must be obtained through dietary intake. Furthermore, four acids with antioxidant properties were identified: cannabidiolic acid (approximately 19.82% of all identified compounds), cinnamic acid (1.06%), gallic acid (1.03%), and cannabidivarinic acid (0.56%). Moreover, all four of these acids also possess antibacterial properties, as does another identified acid: oxalic acid (0.70%) [28,29,30,31].

### 3.4. Antimicrobial Activity

Hemp inflorescence extracts obtained using 2% Triton X-100 had the highest antimicrobial activity—they showed an effect on all tested strains of microorganisms (Table 3). *C. albicans* was sensitive only to hemp extract obtained using 2% Triton X-100, under the influence of which a growth inhibition zone of (3.5 ± 1.5) mm was formed. This extract most strongly inhibited the growth of *P. aeruginosa* (22.0 ± 1.5) mm, *B. subtilis* (19.5 ± 1.5) mm, *B. megaterium* (15.0 ± 4.7) mm, *B. cereus,* and *L. monocytogenes* (14.5 ± 0.5) mm. *P. aeruginosa* and *B. subtilis* were also sensitive to aqueous hemp extracts: 7.0 ± 2.0 and 7.0 ± 1.0 mm, respectively, while aqueous hemp extract had not shown an antibacterial effect on other tested bacterial strains.

Exposure of test bacteria to H_2_O hemp extract produced growth inhibition zones of less than 4 mm in plates containing cultures of *S. aureus*, *B. megaterium,* and *S. typhymurium* bacteria.

Seventy percent (70%) MeOH hemp extract had a high antibacterial effect on 7 tested bacterial strains, including *B. cereus*, *B. subtilis*, *B. megaterium*, *L. monocytogenes*, *E. faecalis*, *M. luteus,* and *S. enteritidis*; the size of the inhibition zones for all these microorganisms was quite large and very similar—14.5 mm; only the growth inhibition zone of *B. cereus* is slightly larger—(19.5 ± 0.5) mm.

## 4. Discussion

Results regarding TFC showed that MeOH demonstrated the best extraction affinity, with 83% and 20% more TFC as compared to water and Triton X-100, respectively (Figure 1). Likewise, for TPA contents, MeOH yielded 86% and 67% more against Triton X-100 and water, respectively (Figure 2a). In the same way, for RSA, MeOH again yielded 5-folds and 40% more than Triton X-100 and water (Figure 2b). The higher MeOH extraction efficiency could be due to its polarity and alcoholic potential to extract both hydrophilic and lipophilic substances [32]. Moreover, alcoholic solvents have adaptability in a wide range of temperatures, such as sub-zero to boiling point [33]. Our results are in line with other authors’ works [32,34], who compared different solvents (water, ethanol, methanol, acetone, hexane, and dichloromethane) for their efficiency in extracting antioxidants from two different plant species. In the results, they stated that MeOH proved to be the best one, while hexane remained the least effective one.

Results regarding TPC showed that Triton X-100 showed the best extraction affinity with 4-folds and 33% more TPC as compared to water and MeOH, respectively (Figure 1). The good extraction efficiency of non-ionic Triton X-100 could be attributed to its structural property, which associates with different plant compounds and mediates their solubilization [20]. Our results are in line with the experiment [20] who observed the significant potential of Triton X-100 for the extraction of phenolic compounds from the flower part of the dandelion.

Moreover, Figure 2 demonstrates the correlation of RSA with TPA contents, which indicates that the presence of TPA in hemp inflorescence is responsible for improving the antiradical scavenging activity of the hemp plant. This finding relates to an experiment in which higher polyphenolic compounds reflected higher antioxidant activity in *T. officinale* [20].

Results regarding cannabinoids composition in hemp inflorescence showed that methanolic extraction showed maximum affinity for CBD contents with a value of 2.50%, followed by CBC with a value of 0.330% from a total of 3.5% cannabinoids (Table 1). Literature analysis confirmed the presence of diverse cannabinoids in hemp plants, such as CBG, CBD, CBC, CBL, CBE, and CBN [35]. Obtained results correspond to different experiments that describe the cannabinoids composition in different hemp parts using methanolic extraction [14,36].

Furthermore, Table 2 represents the diversity of acids in hemp inflorescence. The data indicate that identified acids have varying degrees of predominance, with oxalic acid having the lowest amount and cannabidiolic acid having the highest, with an average percentage of 0.70% and 19.82%, respectively, of all identified compounds. As expected, cannabidiolic and cannabidivarinic acids were found in hemp inflorescence, which make up approximately 20% of all identified acids, which is consistent with earlier studies [30]. It should also be noted that these acids demonstrate antibacterial properties; recently, researchers [37] determined that these acids have the potential to damage the cell wall and membrane of *E. coli*. For this reason, hemp inflorescences, which contain relatively large amounts of acids with antimicrobial properties, are a promising raw material for the future food, pharmaceutical, and beauty industries [37].

Results regarding antimicrobial activity described that hemp inflorescences extracted with 2% Triton X-100 performed best as compared to 70% MeOH and then H_2_O. Previous studies confirmed the effect of different extraction solvents on antimicrobial activities against different microbial strains [38]. It is documented that hemp floral parts possess the potential for antimicrobial activity against various microbial strains [38]. Recently, researchers described that the antimicrobial potential of hemp inflorescences is due to the presence of phytochemicals such as terpenoids, cannabinoids, TPC, TF, and RSA [39,40]. In our case, 2% Triton X-100 showed stronger antimicrobial affinity against all microbial strains. It is attributed to the presence of TPC and TF, which were significant when extracted with 2% Triton X-100 (Figure 1) [39]. Our results are in accordance with the experiment [22], which tested different solvents (acetone and methanol) in the extraction of the hemp plant. The authors assessed the antimicrobial activity of hemp plants, which was attributed to the TPC concentration in the respective solvent.

## 5. Conclusions

This study was conducted to determine the effects of different polarity solvents on biologically active compound recovery from industrial hemp inflorescences and to investigate the extract’s antimicrobial activity against Gram-negative and Gram-positive bacterial strains and yeast-like fungi. The obtained results revealed that MeOH showed a better affinity for the recovery of TFC, TPA, and RSA in comparison to Triton X-100 and water. Where 2% Triton X-100 was the best solvent for TPC recovery. Likewise, results regarding antimicrobial activity demonstrated 2% Triton X-100 extracts with the highest antimicrobial activity. The aqueous extracts of hemp inflorescence showed antibacterial activity against *S. aureus*, *B. cereus*, *B. subtilis*, *L. monocytogenes*, *B. megaterium, P. aeruginosa*, and *S. typhymurium*. The subject findings suggest the significant comparative potential of extractants for obtaining phytochemicals from plant parts along with antimicrobial activity against various microbial strains. Among these extractants, 2% Triton X-100 could be suggested as the best extractant for the recovery of TPC, along with its potential antimicrobial effects. Thereafter, these findings make hemp a prospective plant based on environmentally friendly technologies for various industries.

## Figures and Tables

**Table 1:** The concentration of cannabinoids determined in methanolic extracts of industrial hemp inflorescences.

	CBDVA	CBL	CBD	CBC	CBN	CBG
Hemp inflorescences	**%**					
	0.094 ± 0.0890	0.024 ± 0.0009	2.50 ± 0.1048	0.330 ± 0.1217	0.022 ± 0.0000	0.134 ± 0.0093

Note: CBDVA—cannabidivarinic acid; CBL—cannabicyclol; CBD—cannabidiol; CBC—cannabichromene; CBN—cannabinol; CBG—cannabigerol.

**Table 2:** Organic acids concentration determined in industrial hemp inflorescences; min: minimum value; max: maximum value; mean: average value; CV: coefficient of variance. Results are presented as % of all identified compounds.

Phenolic Acids	Min	Max	Mean	CV
Cinnamic acid, TMS derivative	0.48	2.19	1.06	82.33
Gallic acid, 4 TMS derivative	0.88	1.13	1.03	11.75
Other Organic Acids				
Monoamidomalonic acid, 3 TMS derivative	1.00	5.11	2.85	68.63
Palmitic acid, TMS derivative	8.79	11.88	10.31	10.54
Arachidonic acid, TMS derivative	0.24	1.56	0.77	83.96
Arachidic acid, TMS derivative	0.64	0.89	0.76	16.49
Linoleic acid, TMS derivative	13.01	24.91	18.89	24.16
Alfa-linolenic acid, TMS derivative	9.47	17.13	12.92	25.13
Oleic acid, TMS derivative	1.71	3.25	2.76	21.84
Stearic acid, TMS derivative	3.24	5.14	4.36	15.78
Cannabidivarinic acid, TMS derivative	0.52	0.60	0.56	7.14
Cannabidiolic acid, TMS derivative	15.43	24.99	19.82	21.63
Oxalic acid, TMS derivative	0.38	1.18	0.70	62.60
Malonic acid, TMS derivative	0.11	1.46	1.04	50.89

**Table 3:** The antimicrobial activity of industrial hemp inflorescences extracts.

No	Microorganism Strain	Strain Identification Number	Width of Growth Inhibition Zone, mm (Mean ± Standard Deviation)		
			H_2_O	70% MeOH	2% Triton X-100
1	*S. aureus*	ATCC 25923	4.0 ± 1.0	0.0 ± 0.0	12.0 ± 3.0
2	*B. cereus*	ATCC 11778	4.5 ± 1.5	19.5 ± 0.5	14.5 ± 1.0
3	*B. subtilis*	ATCC 6633	7.0 ± 2.0	14.5 ± 0.5	19.5 ± 1.5
4	*L. monocytogenes*	ATCC 13932	5.5 ± 1.5	14.5 ± 1.0	14.5 ± 0.5
5	*B. megaterium*	ATCC 33085	3.0 ± 1.5	14.5 ± 0.5	15.0 ± 4.7
6	*E. faecalis*	ATCC 19433	0.0 ± 0.0	14.5 ± 2.0	4.5 ± 0.5
7	*M. luteus*	ATCC 9341	0.0 ± 0.0	14.5 ± 1.0	9.5 ± 1.0
8	*S. enteritidis*	ATCC 13076	0.0 ± 0.0	14.5 ± 0.5	4.5 ± 0.5
9	*E. coli*	ATCC 8739	0.0 ± 0.0	0.0 ± 0.0	10.0 ± 2.5
10	*P. aeruginosa*	ATCC 10145	7.0 ± 1.0	0.0 ± 0.0	22.0 ± 1.5
11	*S. typhymurium*	ATCC 14028	2.0 ± 1.0	0.0 ± 0.0	2.5 ± 2.0
12	*C. albicans*	ATCC 10231	0.0 ± 0.0	0.0 ± 0.0	3.5 ± 1.5

## Data Availability

The data that support the findings of this study are openly available on request.
